# Highly pathogenic avian influenza A/H7N3 in great-tailed grackles (*Quiscalus mexicanus*) in the Altos de Jalisco region of Mexico

**DOI:** 10.1099/jmmcr.0.001461

**Published:** 2014-12-01

**Authors:** R Navarro-López, L. F Vázquez-Mendoza, C. L Villarreal Chávez, M. T Casaubon y Huguenin, M. A Márquez Ruiz

**Affiliations:** ^1^​ Animal Health General Directorate, Animal & Plant Health, Food Inspection and Food Safety National Services (SENASICA), Secretariat of Agriculture, Livestock, Rural Development, Fisheries and Food (SAGARPA), Mexico City, Mexico; ^2^​ Faculty of Veterinary Medicine, National Autonomous University of Mexico (UNAM), Mexico City, Mexico

**Keywords:** avian influenza, emerging infectious diseases, H7N3 HPAI, *Quiscalus mexicanus*

## Abstract

**Introduction:**

In June 2012, the presence of a severe highly pathogenic avian influenza (HPAI) outbreak produced by an influenza type A, subtype H7N3 (A/H7N3) virus was reported in Mexico, which significantly affected the region of Los Altos de Jalisco, the most important table-egg production zone in Mexico.

**Case presentation:**

To the best of our knowledge, this is the first report describing the occurrence of infection in wild endemic birds, and particularly in the great-tailed grackle (*Quiscalus mexicanus*), by an HPAI A/H7N3 orthomyxovirus, during the avian influenza epizootic, which occurred in June–October 2012, in the Los Altos region of Jalisco, Mexico, a highly significant poultry area. The great-tailed grackle population has increased significantly due to intense agricultural and livestock farming expansion throughout North and Central America and northern South America, in diverse ecological systems. The great-tailed grackle’s infectious/epidemiological role is unknown, as is its role as the avian influenza virus reservoir and as disseminator of other infectious diseases.

**Conclusion:**

Because of the huge impact that avian influenza virus has on food production, on the economic activity of the affected areas and on the public health of animal and human populations, it is necessary to further investigate the significance of a wild population existing in the vicinity of industrial poultry farms and backyard poultry operations.

## Introduction

In June 2012, the presence of a severe highly pathogenic avian influenza (HPAI) outbreak produced by an influenza type A, subtype H7N3 (A/H7N3) virus was reported in Mexico, which significantly affected the region of Los Altos de Jalisco, the most important table-egg production zone in Mexico (Unión Nacional de Avicultores, 2013).

After a 17-week epidemiologically silent period in January 2013, the epizootic remerged in a second epizootic wave, spreading through the Aguascalientes, Guanajuato, Tlaxcala and Puebla regions, affecting both industrial and backyard poultry such as broilers, layer hens, breeders, fighting cocks and quail flocks.

The control measures that were implemented by the Mexican official veterinary services were stamping out, sanitary disposal, cleanliness, disinfection, biosecurity actions and the movement live and dead animals, poultry products and by-products. In addition, a temporary vaccination campaign was implemented with an oil-inactivated vaccine.

The Los Altos de Jalisco region consists of 46 counties covering 19 599 km^2^, and is the most important table-egg production zone of the country. Forty-two counties were declared under internal quarantine according to the official law by the Secretariat of Agriculture, Livestock, Rural Development, Fisheries and Food (SAGARPA), on 8 June 1998 (Diario Oficial de la Federación, 1996). In this region, there exist a huge number of poultry farms and a significant presence of both natural and artificial bodies of water, where migratory wild aquatic birds following the Pacific migratory route spend the autumn and winter seasons feeding and reproducing in and where they cohabitate for 4–5 months (Western Hemisphere Shorebird Reserve Network, 2009). Likewise, there exists a huge endemic population of native wild birds that live in close proximity to the industrial poultry farms searching for food, despite the fact that poultry houses are closed and protected against the entrance of wild birds.

During epidemiological weeks 31 and 36 in 2012, Tepatitlán de Morelos, Jalisco, was the county where most of the outbreaks by the HPAI A/H7N3 virus were reported in layer hens and broiler breeders flocks. The official human health services were informed about two cases of conjunctivitis without fever or respiratory symptoms caused by HPAI A/H7N3 virus infection in humans associated with exposure to infected poultry (Barrera–Badillo *et al.*, 2012). Mortality was also observed in feral birds known as great-tailed grackles or Mexican zanate (*Quiscalus mexicanus*). This bird is classified within the Passeriform order belonging to the Icteridae family (CONABIO, 2007). Great-tailed grackles are originally from the Gulf of Mexico rim. It is known that grackles were introduced to central Mexico during the time of Ahuitzotl, the eighth Aztec emperor (1486 and 1502) (Gurrola Hidalgo *et al.*, 2009). Grackles currently have a wide distribution in Mexico, in 21 states of the USA and in three provinces in southern Canada (Wehtje, 2003; LeBaron, 2008). Likewise, grackles have been observed in all Central American countries and in Colombian, Venezuela, Ecuador and Peru (IUCN, 2013). The reason for this wide geographical distribution is the grackle’s adaptability and tolerance to urban environments. Other sources of food are associated with agriculture and livestock activities, where there are plentiful grains and other kinds of food, which provides grackles with a continuous source of feeding (Instituto Nacional de Biodiversidad, 2004). Other common places where grackles obtain food are urban garbage sites and from waste food on the streets. Studies have shown that grackles are opportunists, as they can feed on vertebrates and invertebrates, such as small crustaceans and other marine animals, as well as on grains and fruits. Grackles have a preference for water bodies, lagoons, dams, canals, creeks and swamps, where they find a rich source of food. It has been shown that *Q. mexicanus* can be carriers of *Salmonella* (Minott Picado & Caballero Castillo, 2007) and that they can be antibody positive to low pathogenic avian influenza H5 virus and Newcastle disease virus (Escobar, 2011). In Mexico, this species has been involved with transmission of West Nile virus (Guerrero–Sánchez *et al.*, 2011).

## Case report

**Fig. 1.**
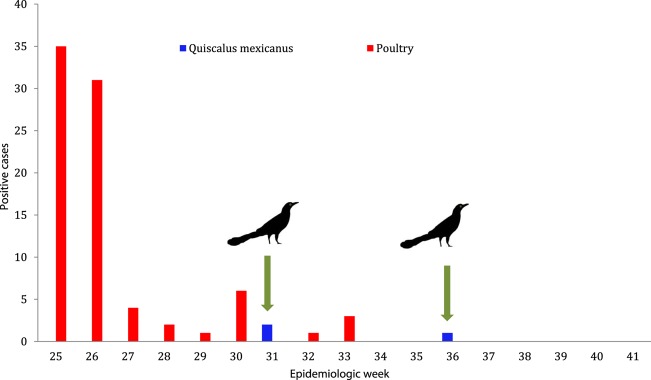
Virus activity in poultry and wild birds during the HPAI A/H7N3 outbreak in Los Altos de Jalisco, Mexico, in 2012. Source: Animal Health General Directorate, SENASICA.

During the 2012 avian influenza outbreak in Jalisco, on weeks 31 and 36, the presence of sick and dead wild birds was identified in a public park of Tepatitlán, Unidad Deportiva Morelos, located at geographical co-ordinates 20° 48′ 17.22″ N, 102° 45′ 48.12″ W, by the veterinary sanitary officers of the Animal & Plant Health, Food Inspection and Food Safety National Services (SENASICA) (Fig. 1). During the epidemiological research that was carried out, it was reported that the affected birds belonged to the species *Q. mexicanus*. According to the clinical history (case nos CPA-16126-12 and CPA-16172-12), 10 birds were found. Eight were dead, one was ill and the other could not fly. Dead birds were not sampled because they were already rotten. The sick bird presented head oedoema, torticollis and diarrhoea. Both living birds were euthanized in order to carry a post-mortem examination. No macroscopic lesions were reported. Organ samples and tracheal and cloacal swabs were sent to the official animal health diagnostic laboratories of SENASICA in Jalisco and in Mexico City.

**Fig. 2.**
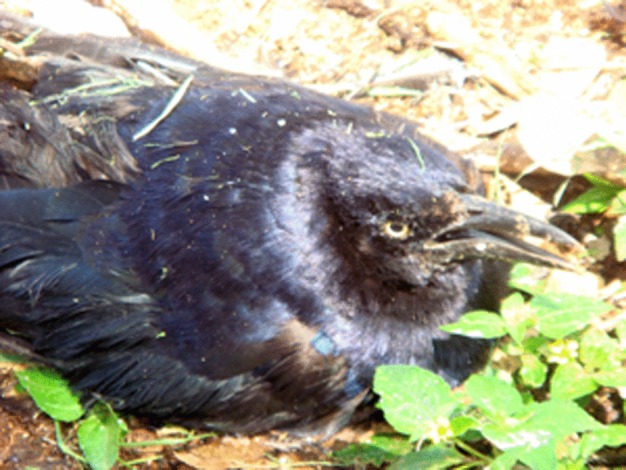
An adult great-tailed grackle (*Q. mexicanus*), which was prostrated and unable to fly, showing incoordination, gasping and dark brown diarrhoea.

On epidemiological week 36 of 2012, during a new official search at the same park in Tepatitlán, where the HPAI cases were reported 5 weeks previously, another ill adult grackle was detected, which was prostrated and unable to fly, showing incoordination, gasping and dark brown diarrhoea (Fig. 2). Another dead bird was also collected from the football field and identified as a bronzed cowbird (*Molothrus* sp.) in the Passeriform order (CONABIO, 2008). Both birds were taken, under strict biosecurity measures, to the Animal Pathology Laboratory in El Salto, Jalisco, in order carry the post-mortem examination and to take organ samples for virological and histopathological tests. The grackle died during transportation.

After necropsy, 10 organ samples were sent in 10 % formalin (brain, lung, trachea, liver, heart, proventriculus, gizzard, duodenum, intestine and pancreas) to the Avian Pathology Laboratory of the Veterinary Medicine Faculty/National Autonomous University of Mexico (case no. 1056-12).

Employees and gardeners working at the park stated that it had been common to find sick or dead grackles during the previous 6 weeks.

In the back part of the park is a river where there are Montezuma cypress trees (*Taxodium mucronatum*) and Benjamin’s ficus trees (*Ficus benjamina*) where grackles build their nests. In the ficus trees and on the ground below them were fecal material and feathers, indicating that the birds live in these trees.

During this epidemiological study of the perifocal zone, the presence of two poultry farms, one layer farm (62 000 hens) and one broiler farm (295 000 chickens) was detected within a 1-mile radius. The layer farm animals were vaccinated for avian influenza A/H7N3 virus, but the birds in the broiler farm were not vaccinated for avian influenza.

As result, an HPAI A/H7N3 virus was isolated in chicken embryos inoculated with organs and the cloacal and tracheal swabs (case nos CPA-16126-12 and CPA-16172-12), from samples taken during epidemiological week 31. Viral isolation, identification, sequencing and intravenous pathogenicity index (values of 2.96–3.0) studies were carried out, confirming that the infection was caused by the HPAI A/H7N3 virus. The isolated virus was identical to that isolated from layers hens during the HPAI outbreak.

Post-mortem examination in cases CPA-19955-12 and CPA-1056-12 from week 36 showed congestion and suffusions in the heart and enteritis in the grackle. The rest of the organs did not show macroscopic or microscopic lesions. The cowbird died due to a severe skull encephalic trauma.

Histopathology examination of the grackle bird organs revealed non-suppurative encephalopathy, severe fatty hepatitis and moderate suppurative enteritis with moderate presence of coccidia. The rest of the organs did not present significant pathological changes.

Histopathological study of the organs taken from the cowbird showed a neoplasic lymphoid process in the liver and proventriculus. Intestinal obstruction by nematodes and the presence of coccidia in the duodenum were observed.

Frozen organ samples were studied to determine which organs contained viruses. Official SENASICA BSL2 and BSL3 laboratories confirmed the presence of HPAI A/H7N3 virus isolation from the grackle’s heart. Virology was negative in the case of the cowbird.

Broilers in the Los Altos area were not vaccinated but did not become ill during this first epidemiological wave.

## Discussion

It was confirmed that, during epidemiological weeks 31 and 36, great-tailed grackles became sick and died due to an infection produced by HPAI A/H7N3 virus. Although it was not possible to prove that the layer hens in the neighbouring farms were the direct source of this infection, it is well known that feral birds, and in this particular case grackles, can fly around for a number of kilometres, feeding close to the farms where they get infected. Another infection source could have been the permanent trash deposits where the grackles feed.

Wild birds have been involved in infectious disease transmission, either as reservoirs or as mechanical vehicles of infectious and parasitical diseases (Hénaux *et al.*, 2012). It has also been shown that migratory and resident native wild birds can carry low pathogenic avian orthomyxovirus (García Espinoza, 2008). *Q. mexicanus* and other wild native birds are common visitors of poultry farms.

We consider that the transmission chain mechanism was closed, because it contained all the elements required to assure the transmission of an infectious agent from ill to healthy birds (in this case, over at least 5 weeks).

At the present time, the HPAI A/H7N3 virus is still circulating in four states of Mexico, and therefore it is necessary to continue investigating the infection and epidemiological role of grackles and other wild native birds living in the surroundings of poultry farms as reservoirs and transmitters of infectious diseases, taking into account the fact that avian influenza depends on virus prevalence in the host reservoir species (Garamszegi & Møller, 2007). It is important to understand the role and significance of this wild avifauna in order to design strategies for the control and eradication of avian influenza in Mexico.

## Abbreviations

HPAI: highly pathogenic avian influenza
SENASICA: Animal & Plant Health, Food Inspection and Food Safety National Services.
